# B cells control lupus autoimmunity by inhibiting Th17 and promoting Th22 cells

**DOI:** 10.1038/s41419-020-2362-y

**Published:** 2020-03-03

**Authors:** Ji Yang, Xue Yang, Luman Wang, Ming Li

**Affiliations:** 1 Department of Dermatology, Zhongshan Hospital, Fudan University, Shanghai, China; 2 Division of Rheumatology, Huashan Hospital, Fudan University, Shanghai, China; 3 Institute of Rheumatology, Immunology and Allergy, Fudan University, Shanghai, China; 4 Department of Immunology, Basic Medical School, Fudan University, Shanghai, China

**Keywords:** Autoimmunity, Autoimmune diseases

## Abstract

B cells exert immunosuppressive effects and offer therapeutic potential for systemic lupus erythematosus (SLE), but the mechanism remains unclear. Here we analyzed the B cell regulation of Th17/Th22 cell differentiation in lupus and found that α-IgM- and α-CD40-activated B cells could inhibit Th17 and promote Th22 cell differentiation from naive T cells under Th17 cell culture conditions. B cell-induced Th22 cells demonstrated immunosuppressive effects and could decrease renal endothelial cell apoptosis in vitro. Moreover, activated B cell infusion relieved lupus injuries via IL-22 production in vivo. Mechanically, activated B cells affected Th17/Th22 cell differentiation by non-contact TNF-α secretion and mTOR activation. Finally, activated B cells could affect Th17/Th22 cell differentiation in human peripheral blood T cells. These data suggest that activated B cells might attenuate lupus autoimmunity by inhibiting Th17 but promoting Th22 cell differentiation, supporting B cell activation as a promising therapeutic for the treatment of lupus.

## Introduction

Systemic lupus erythematosus (SLE) is a common autoimmune disease that involves multiple organ systems, occurring in 20–150 for every 100,000 people^1^. The pathogenesis of SLE remains unclear, although an imbalance within the immune system has been implicated. Therefore, it is necessary to investigate the immunological mechanisms of lupus to provide greater clarity and knowledge toward viable treatments.

In the disease microenvironment, B cells can be activated and induced into regulatory immune cells that affect disease progression^2–5^. Activated B cells produce cytokines such as interleukin (IL)-10, transforming growth factor (TGF)-β, and tumor necrosis factor (TNF)-α^6,7^, and the absence of B cells exacerbated disease symptoms in models of lupus, experimental autoimmune encephalomyelitis, and collagen-induced arthritis^3,4,8–10^. Previous studies have shown B cell dysregulation in SLE patients and MRL/lpr mice^10–12^, especially with immunosuppressive functions^13^. These immunosuppressive effects could exert therapeutic benefits against lupus^14^, but how B cells are regulated is not fully understood.

The T helper 17 (Th17) cell lineage, a lineage of effector CD4^+^ T cells characterized by IL-17 production^15,16^, is associated with the pathogenesis of autoimmune diseases, including SLE^17–20^. Our studies, as well as others, have shown that Th17 cells were expanded in SLE similar to inflammatory tissue injuries and autoantibody production^17,20,21^. Thus Th17 cell inhibition could help to relieve lupus autoimmune injuries. IL-22-producing CD4^+^ T (Th22) cells are a new subset of CD4^+^ T cells with immunosuppressive capabilities and differentiated from naive T cells through TNF-α and IL-6^22,23^. Although IL-22 can be produced during Th17 cell differentiation^24,25^, the reciprocal differentiation of Th17 by Th22 cells in lupus autoimmunity is not clear.

Because the potential for B cells to affect Th17 and Th22 cell differentiation has not been reported, we have analyzed the mechanisms and potential therapeutic role through which B cells affect Th17 and Th22 cell differentiation in the treatment of lupus.

## Materials and methods

### Mice treatment

CD45.2^+^C57BL/6 (B6) and lupus-prone MRL/lpr mice were purchased from the Shanghai Laboratory Animal Center (Chinese Academy of Sciences). CD45.1^+^ mice were purchased from The Jackson Laboratory (Bar Harbor, ME). Animal studies were approved by the Institutional Animal Care and Use Committee of Zhongshan Hospital, Fudan University. Mice were maintained under pathogen-free conditions. Twelve-week-old MRL/lpr mice were randomized into four groups, and the mice were injected intravenously with 10 × 10^6^ ex vivo-expanded B cells or phosphate-buffered saline (PBS) control with or without 2.5 µg/g anti-IL-22 antibody (Thermo Fisher Scientific, Waltham, MA, USA) weekly for 4 weeks. The animal study is not blinding. Urine was collected for the first 24 h and assayed to detect protein by Coomassie brilliant blue according to the manufacturer’s instructions (Nanjing Jiancheng, China). Four weeks after treatment, MRL/lpr mice were sacrificed and the spleens and inguinal lymph nodes were collected and weighed. The percentages of CD4^+^IL-17^+^ Th17 cells and CD4^+^IL-22^+^ Th22 cells in the spleens were analyzed by flow cytometry, including retinoic acid–related orphan receptor γt (RORγt) and c-Maf intracellular expression. Kidney tissues were fixed for assessment.

### Naive CD4^+^ T and B cell isolation and differentiation

For Th17 cell differentiation, naive CD4^+^ T cells were purified from the spleens of B6 mice using the naive Mouse CD4 Cell Kit (StemCell Technologies, Vancouver, BC, Canada). Sorted naive CD4^+^ T cells were cultured under Th17 cell culture conditions with 2 µg/mL anti-CD3, 2 µg/mL anti-CD28, 1 ng/mL TGF-β, 50 ng/mL IL-6, 10 ng/mL IL-1β, 5 ng/mL IL-23, 10 µg/mL anti-IL-4, 10 µg/mL anti-IFN-γ, and 10 µg/mL anti-IL-2 (all from Thermo Fisher Scientific) for 5 days.

For Th1 cell differentiation, naive CD4^+^ T cells were purified from the spleens of B6 mice using the naive Mouse CD4 Cell Kit (StemCell Technologies, Vancouver, BC, Canada). Sorted naive CD4^+^ T cells were cultured under Th1 cell culture conditions with 2 µg/mL anti-CD3, 2 µg/mL anti-CD28, 20 ng/mL IL-2, 20 ng/mL IL-12, and 10 µg/mL anti-IL-4 (all from Thermo Fisher Scientific) for 5 days.

For regulatory T (Treg) cell differentiation, naive CD4^+^ T cells were purified from the spleens of B6 mice using the naive Mouse CD4 Cell Kit (StemCell Technologies, Vancouver, BC, Canada). Sorted naive CD4^+^ T cells were cultured under Treg cell culture conditions with 2 µg/mL anti-CD3, 2 µg/mL anti-CD28, 5 ng/mL IL-2, and 5 ng/mL recombinant human TGF-β1 for 5 days.

For B cell differentiation, naive B cells were purified from the spleens of B6 mice using the Mouse B Cell Kit (StemCell Technologies). Sorted naive B cells were stimulated for 2 days with 2 µg/mL anti-CD40L and 2 µg/mL anti-IgM (BD Pharmingen, San Diego, CA, USA).

For some experiments, sorted naive B cells were stimulated with 2 µg/mL anti-CD40L and 2 µg/mL anti-IgM (BD Pharmingen) for 2 days and then co-cultured with naive T cells under Th17 cell culture conditions for 5 days. For some experiments, induced B cells were co-cultured with pre-Th17 cells (naive T cells stimulated in Th17 culture conditions for 3 days) for an additional 5 days, after which Th17 and Th22 cell differentiation was analyzed.

For some experiments, sorted naive B cells were stimulated with 2 µg/mL anti-CD40L and 2 µg/mL anti-IgM (BD Pharmingen) for 2 days and then co-cultured with naive T cells under Th1 cell culture condition or Treg for 5 days.

For some experiments, sorted naive B cells were stimulated with 2 µg/mL anti-CD40L and 2 µg/mL anti-IgM (BD Pharmingen) for 2 days, then co-cultured with naive T cells in different transwell chambers under Th17 cell culture conditions with or without 1 µg/mL anti-TNF-α antibody or 5 ng/mL TNF-α (Thermo Fisher Scientific) for 5 days, after which Th17 and Th22 cell differentiation was analyzed.

For some experiments, naive B cells sorted from CD45.1^+^ mice were stimulated with 2 µg/mL anti-CD40L and 2 µg/mL anti-IgM (BD Pharmingen) for 2 days, then co-cultured with naive T cells sorted from CD45.1^+^ mice under Th17 cell culture conditions for 5 days. These induced CD45.1^+^ T cells were sorted and co-cultured with primary renal endothelial cells or naive T or B cells sorted from CD45.2^+^ mice for 3 days under different culture conditions. Renal endothelial cell apoptosis was analyzed by flow cytometry, and T and B cell differentiation was analyzed.

For some experiments, sorted naive B cells were stimulated with 2 µg/mL anti-CD40L and 2 µg/mL anti-IgM (BD Pharmingen) for 2 days, then co-cultured with naive T cells under Th17 cell culture conditions with or without 1 µg/mL anti-TNF-α antibody, 5 ng/mL TNF-α (Thermo Fisher Scientific), 10 μM mammalian target of rapamycin (mTOR) agonist (MHY1485; MedChem Express, USA), or 200 ng/mL rapamycin. mTOR phosphorylation and IL-17 and IL-22 production were then analyzed.

For some experiments, sorted naive B cells from the peripheral blood mononuclear cells (PBMCs) of 3 healthy human donors were stimulated with 2 µg/mL anti-CD40L and 2 µg/mL anti-IgM (BD Pharmingen) for 2 days, then co-cultured with naive T cells sorted from healthy human donor PMBCs under Th17 cell culture conditions for 5 days. Th17 and Th22 cell differentiation was then analyzed. The study protocol was reviewed and approved by the ZhongShan Hospital Research Ethics Committee.

### Flow cytometric analysis

To detect Th17 and Th22 cells, cells were incubated for 5 h with 50 ng/mL phorbol myristate acetate (PMA; Sigma-Aldrich, USA) and 750 ng/mL ionomycin (Sigma-Aldrich) in the presence of 20 μg/mL brefeldin A (Sigma-Aldrich) and then stained with fluorescein isothiocyanate (FITC)-conjugated anti-CD4 for 15 min. Cells were then resuspended in a fixation/permeabilization solution and stained intracellularly with phycoerythrin (PE)-conjugated anti-IL-17, PE-conjugated anti-IL-22, PE-conjugated anti-RORγt, or PE-conjugated anti-c-Maf for 30 min according to the manufacturer’s instructions (Thermo Fisher Scientific). After staining, IL-17^+^, IL-22^+^, RORγt ^+^, and c-Maf^+^ cells were analyzed with a CD4^+^ gate by flow cytometry.

For Th1 cell-related cytokine detection, cells were incubated for 5 h with 50 ng/mL PMA and 750 ng/mL ionomycin in the presence of 20 μg/mL brefeldin A, then stained with FITC-conjugated anti-CD4 for 15 min. Cells were then resuspended in a fixation/permeabilization solution and stained intracellularly with PE-conjugated anti-TNF-α, PE-conjugated anti-IFN-γ, or PE-conjugated anti-TGF-β for 30 min according to the manufacturer’s instructions (Thermo Fisher Scientific). After staining, TNF-α^+^, IFN-γ^+^, and TGF-β^+^ cells were analyzed with a CD4^+^ gate by flow cytometry.

For IL-10 and TGF-β detection in B cells, cells were incubated for 5 h with 50 ng/mL PMA and 750 ng/mL ionomycin in the presence of 20 μg/mL brefeldin A, then surface-stained with FITC-conjugated anti-CD19 for 15 min. Cells were then re-suspended in Fixation/Permeabilization solution (Invitrogen, USA) and stained intracellularly with PE-conjugated anti-IL-10 or PE-conjugated anti-TGF-β for 30 min according to the manufacturer’s protocol (Thermo Fisher Scientific). After staining, IL-10^+^ and TGF-β^+^ cells were analyzed with a CD19^+^ gate by flow cytometry.

For renal endothelial cell apoptosis detection, C57BL/6 mouse primary kidney endothelial cells purchased from Cell Biologics (Chicago, IL, USA) were co-cultured with B cell-induced CD45.1^+^ T cells for 3 days. Then endothelial cell apoptosis was analyzed by the Annexin V Apoptosis Detection Kit (BD Pharmingen). The expression levels of IL-22R (Novus Biologicals, Littleton, CO, USA), CD31, CD45, and ICAM (eBioscience) were detected by flow cytometry.

### Cytokine detection

Sorted T or B cells from mice were cultured with or without B cells, then IL-17, IL-22, TNF-α, interferon (IFN)-γ, TGF-β, IL-10, immunoglobulin M (IgM), and IgG levels in supernatants were determined by enzyme-linked immunosorbent assay (ELISA; all from Thermo Fisher Scientific). Serum double-stranded DNA (ds-DNA) antibody levels in MRL/lpr mice were detected by ELISA (Thermo Fisher Scientific). Sorted T or B cells from healthy human donor PBMCs were cultured with or without B cells, then IL-17 and IL-22 levels in supernatants were determined by ELISA (all from Thermo Fisher Scientific).

### Histopathological assessment

Mouse kidneys were fixed with formaldehyde, embedded in paraffin, and stained with hematoxylin and eosin (H&E). H&E-stained kidney slides were read and interpreted in a blind fashion in which kidneys were graded for glomerular inflammation, proliferation, crescent formation, and necrosis. Interstitial changes and vasculitis were also noted. Scores from 0 to 3 were assigned for each feature, and scores were added to yield a final renal score. For example, glomerular inflammation was graded as follows: 0, normal; 1, few inflammatory cells; 2, moderate inflammation; and 3, severe inflammation.

### Statistical analysis

Quantitative data were expressed as mean ± standard deviation (SD). Differences were determined by unpaired two-tailed *t* test for comparing two groups. For comparing two group values that did not follow Gaussian distribution, the two-tailed Mann–Whitney *U* test was used. All *p* values < 0.05 were considered significant.

## Results

### Activated B cells inhibit Th17 but promote Th22 cell differentiation in vitro

**Fig. 1:**
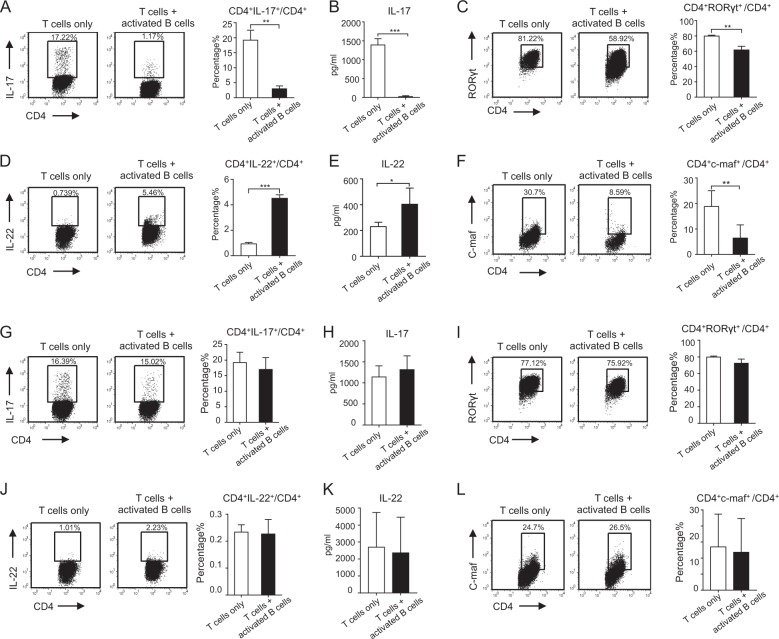
B cells inhibit Th17 but promote Th22 cell differentiation in vitro. Naive B cells isolated from B6 mice were cultured in the presence of α-IgM and α-CD40 for 2 days, then co-cultured with sorted naive T cells under Th17 cell culture conditions (TGF-β, IL-6, IL-1β, IL-23, etc.) for 5 days. **a** CD4^+^IL-17^+^ cells were analyzed by flow cytometry using a CD4^+^ gate (left). The statistics for flow cytometry of CD4^+^IL-17^+^ cells (right). **b** IL-17 in supernatants was analyzed by ELISA. **c** CD4^+^RORγt^+^ cells were analyzed by flow cytometry (left). The statistics for flow cytometry of CD4^+^RORγt^+^ cells (right). **d** CD4^+^IL-22^+^ cells were analyzed by flow cytometry (left). The statistics for flow cytometry of CD4^+^IL-22^+^ cells (right). **e** IL-22 in supernatants was analyzed by ELISA. **f** CD4^+^c-Maf^+^ cells were analyzed by flow cytometry (left). The statistics for flow cytometry of CD4^+^c-Maf^+^cells (right). Sorted naive T cells were pre-cultured in Th17 cell culture conditions for 3 days, then co-cultured with B cells (naive B cells stimulated with α-IgM and α-CD40 for 2 days) for 5 days. **g** CD4^+^IL-17^+^ cells were analyzed by flow cytometry (left). The statistics for flow cytometry of CD4^+^IL-17^+^ cells (right). **h** IL-17 in supernatants was analyzed by ELISA. **i** CD4^+^RORγt^+^ cells were analyzed by flow cytometry (left). The statistics for flow cytometry of CD4^+^RORγt^+^ cells (right). **j** CD4^+^IL-22^+^ cells were analyzed by flow cytometry (left). The statistics for flow cytometry of CD4^+^IL-22^+^ cells (right). **k** IL-22 in supernatants was analyzed by ELISA. **l** CD4^+^c-Maf^+^ cells were analyzed by flow cytometry (left). The statistics for flow cytometry of CD4^+^c-Maf^+^ cells (right). Results shown are representative of three independent experiments. *p < 0.05; **p < 0.01; ***p < 0.001.

B cells are potent negative regulators of inflammation and autoimmunity when activated in vivo and in vitro^7,26^. Here α-IgM- and α-CD40-activated B cells were co-cultured with naive T cells under Th17 cell culture conditions. Compared with the T cell only group, activated B cells inhibited IL-17 production and RORγt expression (a transcription factor of Th17 cells^27^) (Fig. 1a–c). Interestingly, activated B cells promoted CD4^+^IL-22^+^ T cell differentiation and IL-22 secretion even under Th17 cell culture conditions (Fig. 1d, e). In addition, c-Maf, reported as a negative regulator of Th22 cell differentiation^21^, was inhibited by activated B cells (Fig. 1f). These data indicated that activated B cells could inhibit Th17 but promote Th22 cell differentiation. To further analyze whether B cells could reverse Th22 cell differentiation from Th17 cells, activated B cells were co-cultured with established Th17 cells (naive T cells pre-cultured under Th17 culture conditions for 3 days). B cells could neither affect the differentiation of Th17 and Th22 cells nor regulate RORγt and c-Maf expression (Fig. 1g–l). We also determined the effects of activated B cells on other effector T and Treg cell subsets. As Supplementary Fig. 1a–c shows, activated B cells did not affect Th1 or IL-10^+^ and TGF-β^+^ Treg cell differentiation. Collectively, these data indicate that activated B cells might inhibit Th17 and promote Th22 cell differentiation from naive T cells but not differentiated Th17 cells.

### Activated B cell-induced Th22 cells display immunosuppressive effects in vitro

**Fig. 2:**
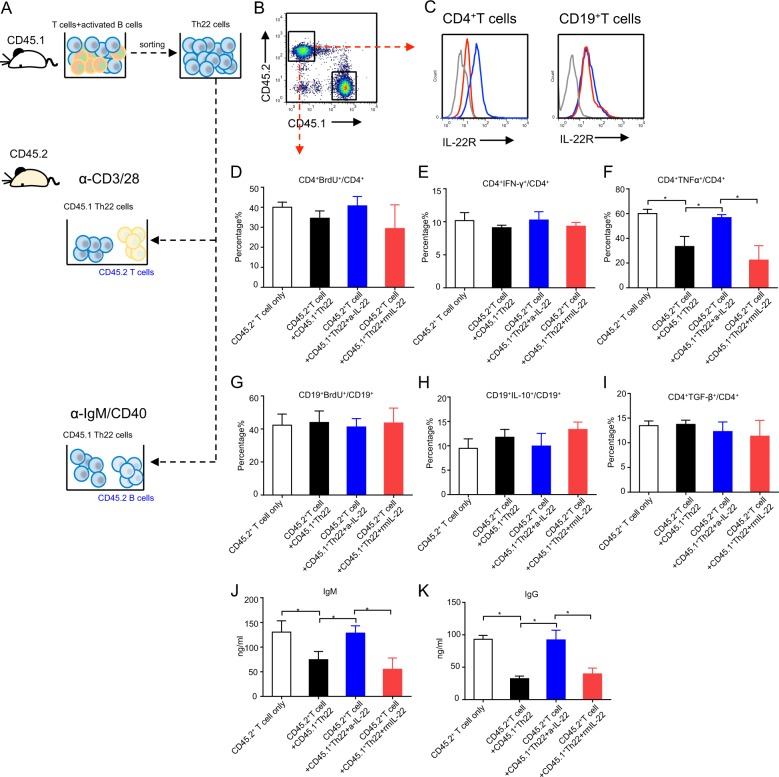
B cell-induced T cells possess immunosuppressive effects via IL-22. **a** The schematic graph show that naive B cells sorted from CD45.1^+^ background B6 mice were cultured in the presence of α-IgM and α-CD40 for 2 days, then co-cultured with sorted naive T cells from CD45.1^+^ B6 mice under Th17 cell culture condition for 5 days. CD45.1^+^ T cells were then sorted and co-cultured with naive T and B cells from CD45.2^+^ B6 mice with or without anti-IL-22 antibodies for additional 5 days. **b** CD45.1^+^ T and CD45.2^+^ T cells were detected by flow cytometry. **c** IL-22R expression in CD45.2^+^ T and CD45.2^+^ B cells was analyzed by flow cytometry. **d** CD45.2^+^ T cells were labeled with BrdU, then cell proliferation was analyzed by flow cytometry. **e** The flow cytometric results of CD4^+^IFN-γ^+^ cells. **f** The flow cytometric results of CD4^+^TNF-α^+^ cells. **g** CD45.2^+^ B cells were labeled with BrdU, then analyzed by flow cytometry. **h** The flow cytometric results of CD19^+^IL-10^+^ cells. **i** The flow cytometric results of CD19^+^TGF-β^+^ cells. **j** IgM in supernatants was analyzed by ELISA. **k** IgG in supernatants was analyzed by ELISA. Results shown are representative of three independent experiments. *p < 0.05.

To analyze the function of activated B cell-induced Th22 cells, induced Th22 cells from CD45.1 background mice were first sorted from the co-culture system on day 5, then co-cultured with naive T and naive B cells sorted from CD45.2 background mice under different culture conditions (Fig. 2a, b). Activated CD4^+^ T cells had increased expression of IL-22R (blue line) compared with naive T cell (red line); B cells also had mildly increased expression of IL-22R (blue line) compared with naive B cell (red line) (Fig. 2c). Induced Th22 cells did not affect T cell proliferation but significantly inhibited the TNF-α production of activated T cells (Fig. 2d–f). Moreover, induced Th22 cells did not affect CD19^+^ B cell proliferation or IL-10 and TGF-β production but inhibited IgM and IgG production (Fig. 2g–k). These inhibitory effects could be reversed by supplementing culture media with anti-IL-22 antibody. The findings suggest that B cell-induced Th22 cells possess immunosuppressive effects via IL-22.

**Fig. 3:**
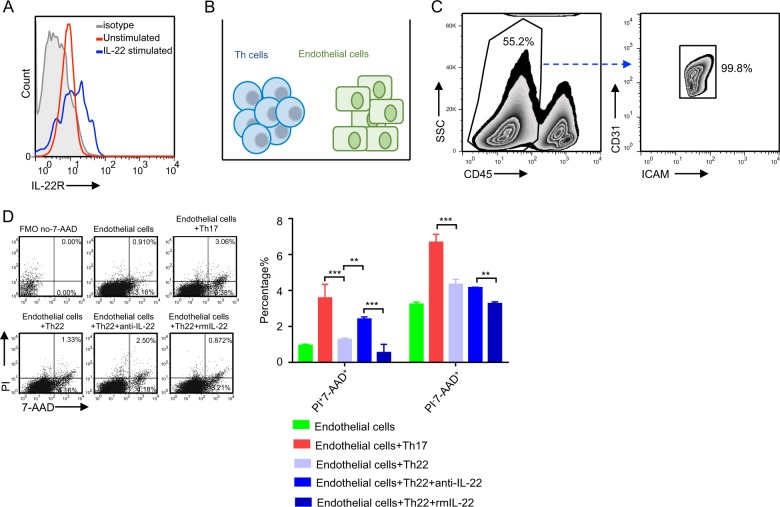
B cells protect renal endothelial cells against apoptosis. **a** The expression of IL-22R in renal endothelial cells with or without IL-22 stimulation was detected by flow cytometry. **b**, **c** Renal endothelial cells were co-cultured with B cell-induced CD45.1^+^ T cells for 3 days. CD31^+^ICAM^+^ endothelial cells were analyzed by flow cytometry. **d** Endothelial cell apoptosis was analyzed by Annexin V Apoptosis Detection kit. Results shown are representative of three independent experiments. **p < 0.01; ***p < 0.001.

Th22 cells can also play a regulatory role in tissue and cellular repair^28,29^. To analyze B cell-induced Th22 cell function, induced Th22 cells were sorted as described above, then co-cultured with mouse primary renal endothelial cells. Renal endothelial cells highly expressed IL-22R and were gated by CD45^−^CD31^hi^ICAM^hi^ (Fig. 3a–c). After 3 days of co-culture, we detected endothelial cell apoptosis, showing that early and late apoptosis were increased when co-cultured with Th17 cells and decreased to control level when cultured with B cell-induced Th22 cells. These effects could be reversed by treatment with anti-IL-22 antibodies in culture media and restored with recombinant mouse (rm) IL-22 (Fig. 3d). These data suggest that activated B cell-induced Th22 cells have protective capabilities over renal endothelial cells.

### Activated B cell-induced Th22 cells rescue lupus in vivo

**Fig. 4:**
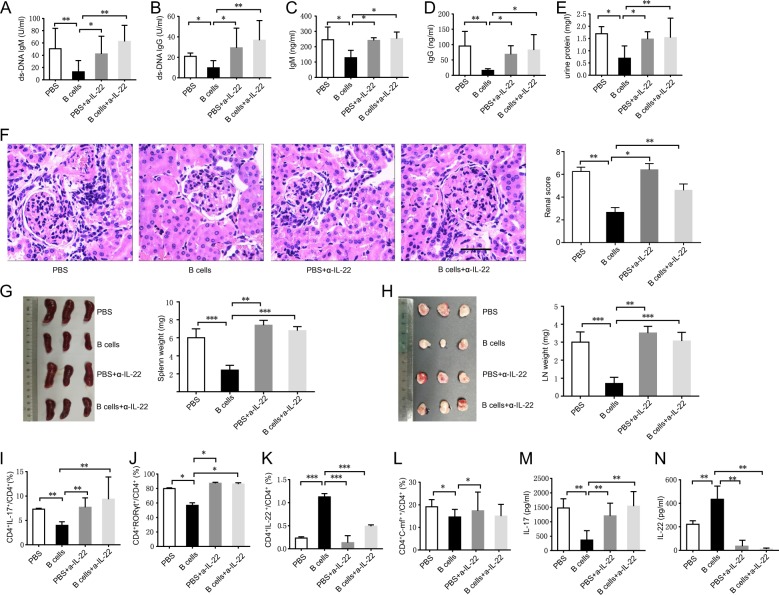
B cells relieve lupus autoimmunity in MRL/lpr mica by IL-22. In all, 10 × 10^6^ ex vivo-expanded B cells or PBS control with or without 2.5 µg/g anti-IL-22 antibodies were injected intravenously into MRL/lpr mice weekly for 4 weeks. **a** Serum anti-ds-DNA antibody IgM level was analyzed by ELISA. **b** Serum anti-ds-DNA antibody IgG level was analyzed by ELISA. **c** Serum IgM level was analyzed by ELISA. **d** Serum IgG level was analyzed by ELISA. **e** Twenty-four-hour urine protein level was analyzed by ELISA. **f** Kidney inflammation was analyzed by H&E staining (left) and the renal scores of MRL/lpr mice (right). **g** Spleen photographs of mice with different treatments (left). Spleen weight of mice with different treatments (right). **h** Inguinal lymph nodes photographs of mice with different treatments (left). Lymph node weights of mice with different treatments (right). **i** CD4^+^IL-17^+^ Th17 cells in the spleen were analyzed by flow cytometry. **j** CD4^+^RORγt^+^ cells were analyzed by flow cytometry. **k** CD4^+^IL-22^+^ Th22 cells were analyzed by flow cytometry. **l** CD4^+^c-Maf^+^ cells were analyzed by flow cytometry. **m** IL-17 level in serum was analyzed by ELISA. **n** IL-22 level in serum was analyzed by ELISA. *n* = 4 for each group. The results shown are representative of three independent experiments. *p < 0.05; **p < 0.01; ***p < 0.001.

In all, 10 × 10^6^ ex vivo-activated B cells or PBS control was injected intravenously into MRL/lpr mice weekly for 4 weeks. Mice injected with B cells displayed an obvious reduction in serum titers of anti-ds-DNA antibody, IgM and IgG, and decreased 24-h urine protein levels (Fig. 4a–e). Treatment with activated B cells relieved lupus autoimmune injuries as observed through reduced kidney inflammatory injuries, decreased renal scores, and the depressed weights of spleens and lymph nodes (Fig. 4f–h), whereas activated B cells injected with anti-IL-22 antibodies abolished these therapeutic effects indicating that activated B cells may exert therapeutic effects via IL-22 (Fig. 4a–h).

Furthermore, treatment with activated B cells inhibited the percentage of CD4^+^IL-17^+^ Th17 cells, decreased intracellular RORγt expression in CD4^+^ T cells of lupus mice spleens, and reduced serum IL-17. However, it also promoted the differentiation of CD4^+^IL-22^+^ Th22 cells, increased serum IL-22, and inhibited intracellular c-Maf expression in CD4^+^ T cells (Fig. 4i–n). Interestingly, anti-IL-22 antibodies injected with B cells reversed B cell regulatory effects on Th17 and Th22 cell differentiation (Fig. 4i–n). These data show that activated B cells could inhibit Th17 but promote Th22 cell differentiation in vivo in lupus-prone mice, supporting the notion that activated B cells could be a promising therapeutic method for treating lupus autoimmunity via IL-22.

### Activated B cells regulate Th17/Th22 differentiation via TNF-α

**Fig. 5:**
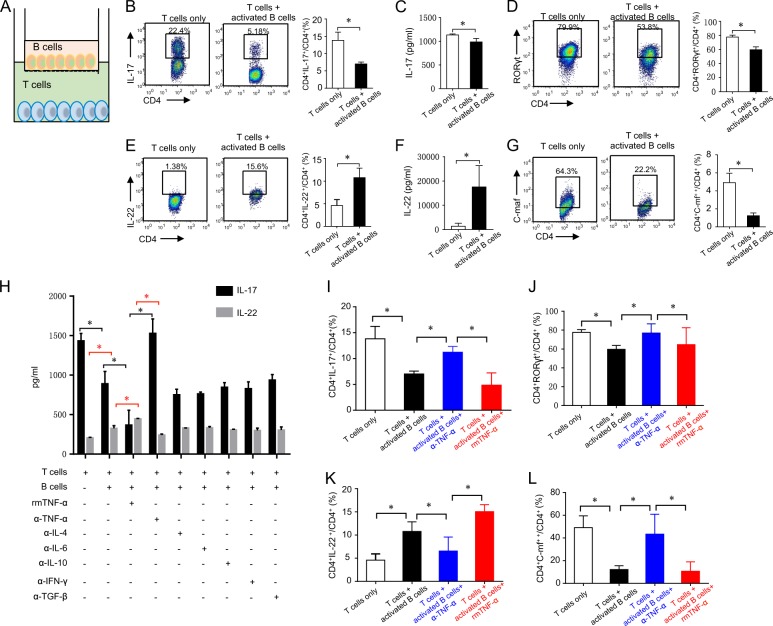
B cells regulate Th17/Th22 differentiation via TNF-α. **a** Naive T cells were co-cultured with B cells (pre-stimulation with α-IgM and α-CD40 for 2 days) in different transwell chambers and under Th17 cell culture condition for 5 days. **b** CD4^+^IL-17^+^ cells were analyzed by flow cytometry (left). The results for flow cytometry of CD4^+^IL-17^+^ cells (right). **c** IL-17 in supernatants was analyzed by ELISA. **d** CD4^+^RORγt^+^ cells were analyzed by flow cytometry (left). The results for flow cytometry of CD4^+^RORγt^+^ cells (right). **e** CD4^+^IL-22^+^ cells were analyzed by flow cytometry (left). The results for flow cytometry of CD4^+^IL-22^+^ cells (right). **f** IL-22 in supernatants was analyzed by ELISA. **g** CD4^+^-Maf^+^ cells were analyzed by flow cytometry (left). The results for flow cytometry of CD4^+^c-Maf ^+^ cells (right). **h** Different cytokines and antibodies were added into cultured media. IL-17 and IL-22 in supernatants were analyzed by ELISA. **i** TNF-α or anti-TNF-α antibodies were added into cultured media. CD4^+^IL-17^+^ cells were analyzed by flow cytometry. **j** TNF-α or anti-TNF-α antibodies were added into cultured media. CD4^+^RORγt^+^ cells were analyzed by flow cytometry. **k** TNF-α or anti-TNF-α antibodies were added into cultured media. CD4^+^IL-22^+^ cells were analyzed by flow cytometry. **l** TNF-α or anti-TNF-α antibodies were added into cultured media. CD4^+^c-Maf^+^ cells were analyzed by flow cytometry. Results shown are representative of three independent experiments. *p < 0.05.

We next explored the mechanism through which activated B cells regulate Th22 differentiation. To identify key factors, activated B cells were co-cultured with naive T cells in different transwell chambers for 5 days. B cells inhibited CD4^+^IL-17^+^ Th17 cell differentiation, IL-17 secretion, and RORγt expression (Fig. 5a–d) while promoting CD4^+^IL-22^+^ Th22 cell differentiation, IL-22 secretion, and inhibiting c-Maf expression (Fig. 5e–g). These data indicate that activated B cells likely inhibit Th17 but promote Th22 cell differentiation without cell contact. We then analyzed the production of cytokines and antibodies produced by activated B cells. Supplementary Fig. 2 shows that α-IgM and α-CD40 induced B cell production of large quantities of TNF-α, IL-10, TGF-β, IFN-γ, and IgM and small quantities of IL-4, IL-6, IgG, and IgA (Fig. S2a–c). In addition to previous studies showing that Th22 cells could be derived from naive T cells in the presence of TNF-α and IL-6^22^, our data demonstrate that B cells also produce TNF-α and other cytokines (Fig. S2a, b). Thus we speculate that B cells might affect Th17/Th22 cell differentiation via TNF-α. We next co-cultured B cells with naive T cells under Th17 cell culture conditions with or without anti-IL-4, anti-IL-6, anti-IL-10, anti-TGF-β, anti-IFN-γ, or anti-TNF-α antibodies or rmTNF-α. The results illustrate that only rmTNF-α and anti-TNF-α antibodies affected IL-17 and IL-22 production (Fig. 5h). Furthermore, neutralization of TNF-α by anti-TNF-α antibodies inhibited Th22 cell differentiation and IL-22 production but promoted Th17 cell differentiation and IL-17 production. Supplementation with rmTNF-α reversed Th17/Th22 cell differentiation and IL-17/IL-22 production, respectively (Fig. 5i–l). All together, these data indicate that activated B cells may affect Th17 and Th22 cell differentiation via non-contact TNF-α secretion.

### Activated B cells regulate T cell differentiation through activation of mTOR

**Fig. 6:**
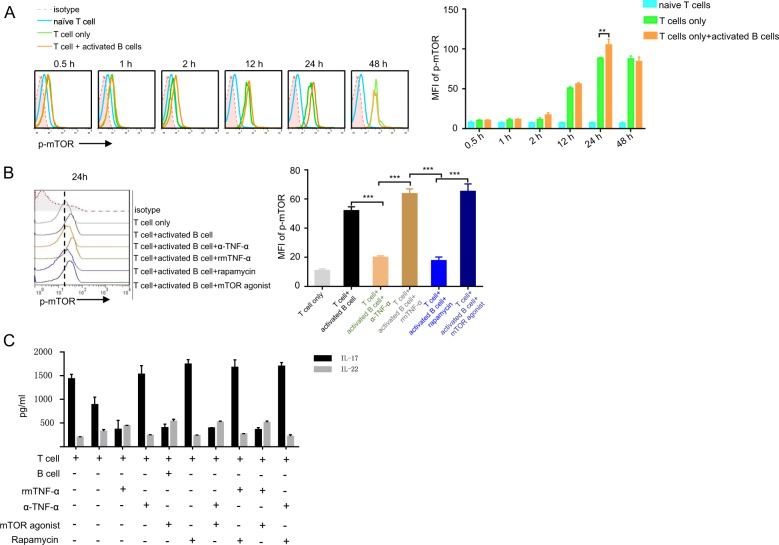
mTOR activation is related to B cell-induced T cell differentiation. **a** Naive T cells were co-cultured with B cells (prestimulation with α-IgM and α-CD40 for 2 days) under Th17 culture conditions over the indicated times, then mTOR phosphorylation was detected by flow cytometry. **b** Naive T cells were co-cultured with B cells (prestimulation with α-IgM and α-CD40 for 2 days) under Th17 culture conditions with or without TNF-α, anti-TNF-α antibodies, rapamycin, or mTOR agonist for 24 h, then mTOR phosphorylation in CD4^+^ T cells was detected by flow cytometry. **c** Naive T cells were co-cultured with B cells (prestimulation with α-IgM and α-CD40 for 2 days) under Th17 culture condition with or without TNF-α, anti-TNF-α antibodies, rapamycin, or mTOR agonist for 48 h. IL-17 and IL-22 in the supernatants were analyzed by ELISA. Results shown are representative of three independent experiments. **p < 0.01; ***p < 0.001.

Although B cells induced Th22 cell differentiation via TNF-α, the biological mechanism remained unclear. mTOR activation has been associated with T cell differentiation^30–33^. Exploring gene expression data in Gene Expression Omnibus, GSE89133 showed that human Th17 cells treated with TNF family member TL1A induced IL-22 secretion, with mTOR activator LAMTOR5 upregulated in the TL1A treatment group after 72-h stimulation^24^ (Supplementary Fig. 3). These data indicated that mTOR activation might be involved in Th22 cell differentiation. To verify this, naive T cells were co-cultured with B cells under Th17 culture conditions over time. Phosphorylation of mTOR was detected across multiple time points in T cells co-cultured with B cells (Fig. 6a). To further analyze the role of mTOR during T cell differentiation, naive T cells were co-cultured with B cells under Th17 cell culture conditions with or without the mTOR agonist MHY1485, mTOR inhibitor rapamycin, TNF-α, or anti-TNF-α antibodies. B cells induced mTOR phosphorylation in T cells, TNF-α and MHY1485 promoted mTOR phosphorylation, and anti-TNF-α antibodies and rapamycin abolished B cell-mediated mTOR phosphorylation (Fig. 6b). Furthermore, when naive T cells were co-cultured with B cells under Th17 cell culture conditions, MHY1485, together with TNF-α, promoted IL-22 production but inhibited IL-17 production, whereas rapamycin and anti-TNF-α antibodies abrogated TNF-α-mediated IL-22 production (Fig. 6c). These data suggest that B cells may promote Th22 cell differentiation through TNF-α and mTOR activation.

### Activated B cells regulate Th17/Th22 cell differentiation in humans

**Fig. 7:**
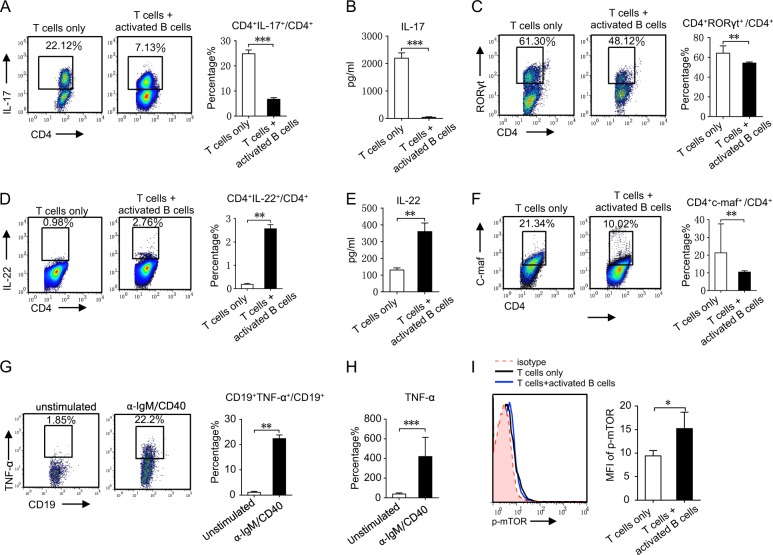
B cells regulate Th17/Th22 differentiation in humans. Naive T cells sorted from the PBMCs of healthy donors were co-cultured with B cells (prestimulation with α-IgM and α-CD40 for 2 days) for 5 days. **a** CD4^+^IL-17^+^ cells were analyzed by flow cytometry (left). The results for flow cytometry of CD4^+^IL-17^+^ cells (right). **b** IL-17 in supernatants was analyzed by ELISA. **c** CD4^+^RORγt^+^ cells were analyzed by flow cytometry (left). The results for flow cytometry of CD4^+^RORγt^+^ cells (right). **d** CD4^+^IL-22^+^ cells were analyzed by flow cytometry (left). The results for flow cytometry of CD4^+^IL-22^+^ cells (right). **e** IL-22 in supernatants was analyzed by ELISA. **f** CD4^+^c-Maf^+^ cells were analyzed by flow cytometry (left). The results for flow cytometry of CD4^+^c-Maf ^+^ cells (right). **g** B cells were cultured with α-IgM and α-CD40 or vehicle control for 2 days. CD19^+^TNF-α^+^ cells were analyzed by flow cytometry (left). The results for flow cytometry of CD19^+^TNF-α^+^ cells (right). **h** TNF-α in the supernatants was analyzed by ELISA. **i** Naive T cells were co-cultured with B cells (prestimulation with α-IgM and α-CD40 for 2 days) under Th17 culture condition for 24 h, then mTOR phosphorylation in CD4^+^ T cells was detected by flow cytometry. Results shown are representative of three independent experiments. *p < 0.05; **p < 0.01; ***p < 0.001.

To further verify the regulatory effects of B cells on Th17 and Th22 cell differentiation in humans, naive T cells isolated from PBMCs were co-cultured with B cells for 5 days (naive B cells were prestimulated with α-IgM and α-CD40 for 2 days). B cells inhibited CD4^+^IL-17^+^ Th17 cell differentiation and intracellular RORγt expression (Fig. 7a, b). Meanwhile, B cells promoted CD4^+^IL-22^+^ Th22 cell differentiation but inhibited c-Maf expression (Fig. 7c, d). B cells also inhibited IL-17 secretion but promoted IL-22 production (Fig. 7e, f). Activated B cells also produced large amounts of TNF-α (Fig. 7g, h). In addition, mTOR phosphorylation was upregulated in T cells co-cultured with B cells (Fig. 7i). These data indicate that B cells could also affect Th17/Th22 cell differentiation in humans.

## Discussion

B cells possess important immunosuppressive effects and play key negative regulatory roles in many autoimmune diseases^3,4,7–9,26^. Previous studies suggest that B cells play a protective role in the autoimmune inflammation injuries of lupus mice^14,34^. However, the mechanisms of its immunosuppressive effects and how B cells alleviate lupus were not clear. In this study, we found that α-IgM- and α-CD40-activated B cells co-cultured with Th17 cells in different transwell chambers and supplemented with rmTNF-α promoted IL-22 and inhibited IL-17 secretion, while blocking TNF-α with TNF-α-neutralizing antibodies had the opposite effects. However, blocking other cytokines did not achieve similar results. Further investigation showed that TNF-α supplementation promoted Th22 and inhibited Th17 cell differentiation, while blocking TNF-α had the opposite effect.

Th17 cells, under investigation for many years, have proven to play important roles in the pathogenesis of autoimmune diseases and become promising therapeutic targets^17–19^. Antibodies against IL-17 secreted by Th17 cells are marketed clinically to treat psoriatic arthritis and ankylosing spondylitis^35,36^. Th17 cells also play key roles in the pathogenesis of lupus; therefore, Th17 cell inhibition may help to alleviate lupus injuries. Here our study demonstrates that activated B cells effectively inhibited Th17 cell differentiation and IL-17 secretion in vitro. In addition, activated B cell infusion therapy also inhibited Th17 cell expansion in lupus mice. Interestingly, the activated B cells inhibited Th17 cell differentiation while promoting Th22 cell differentiation. Activated B cells regulated Th17 and Th22 cell differentiation only when added to the primary culture stage of naive CD4^+^ T cells; they could not regulate Th17 and Th22 cells when naive CD4^+^ T cells were induced to Th17 cells for 3 days. These results suggest that activated B cells can inhibit naive CD4^+^ T cell differentiation into Th17 cells and promote the differentiation into Th22 cells.

mTOR activation plays an important role in lymphocyte activation and proliferation^30–33^. In this study, we found that mTOR was activated in T cells co-cultured with B cells. TNF-α further promoted mTOR activation and induced IL-22 secretion, while TNF-α-neutralizing antibodies inhibited these processes. Since it has been suggested that mTOR activation could be involved in IL-22 secretion from T cells, we confirmed that an mTOR agonist promoted IL-22 secretion, while rapamycin, an mTOR inhibitor, inhibited IL-22 secretion. These data suggest that mTOR activation might be involved in the differentiation of T cells into Th22 cells.

Th22 cells are currently considered to have immunosuppressive effects and play negative regulatory roles in psoriasis, arthritis, and hepatitis^22,37^. Studies have shown that cytokines such as TGF-β, TNF-α, and IL-6 can induce Th22 cell differentiation^22,23^. In this study, a large number of TNF-α secreted by B cells and TGF-β initially added to the culture media might together induce T cells to differentiate into Th22 cells. Although T cells induced by B cells are not all Th22 cells, in vitro functional studies showed that T cells induced by B cells protected endothelial cells against apoptosis, inhibited Th1 cell-related cytokine secretion such as IFN-γ and TNF-α, and also inhibited the B cell secretion of IgM and IgG. Blocking IL-22 with neutralizing IL-22 antibodies alleviated the inhibitory effects of effector T and B cells. It is suggested that T cells induced by activated B cells can play an immunosuppressive role through the secretion of IL-22.

Additional in vivo experiments confirmed that activated B cell infusion could alleviate lupus nephritis, reduce ds-DNA antibody titer and 24 h urinary protein, and inhibit Th17 cell differentiation while promoting Th22 cell differentiation. However, co-injection of B cells with IL-22-neutralizing antibodies significantly weakened the immunosuppressive and therapeutic effects of B cells on Th17 cells, and the percentage of Th22 cells in vivo were also significantly reduced. These results suggest that B cell infusions might exert therapeutic effects in the treatment of lupus mice by inhibiting Th17 cells and promoting Th22 cell differentiation, along with IL-22 playing a key role in B cell protection.

In summary, we define a novel immunoregulatory role for B cells by inhibiting Th17 and promoting Th22 cell differentiation. B cell infusions effectively relieved lupus autoimmunity injuries, suggesting that B cells could be used as an effective therapy in the treatment of lupus.

## Supplementary information

Figure S1

Figure S2

Figure S3

Supplementary figure legend
