# Supplementary figures and images for: B cells control lupus autoimmunity by inhibiting Th17 and promoting Th22 cells
Source: Cell Death Dis. 2020 Mar 3;11(3):164. doi: 10.1038/s41419-020-2362-y (PMC7054432; doi:10.1038/s41419-020-2362-y)

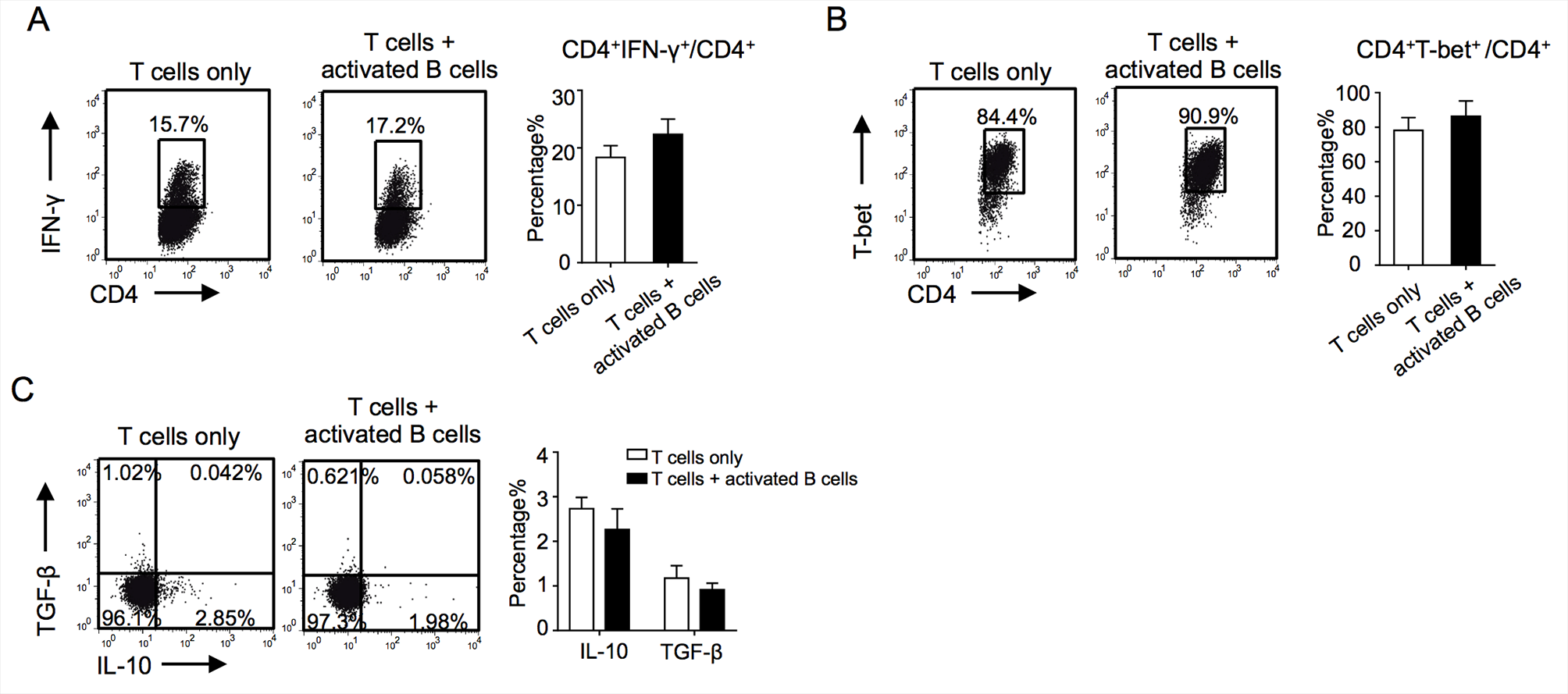

Supplement: Supplementary file 1 — Figure S1 [file 41419_2020_2362_MOESM1_ESM.tif]

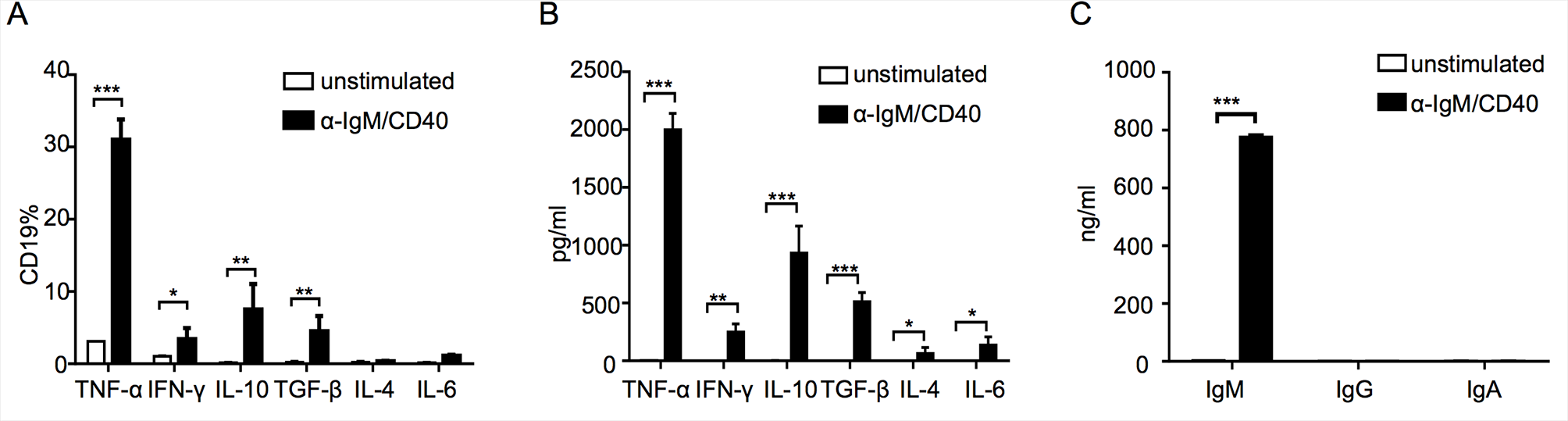

Supplement: Supplementary file 2 — Figure S2 [file 41419_2020_2362_MOESM2_ESM.tif]

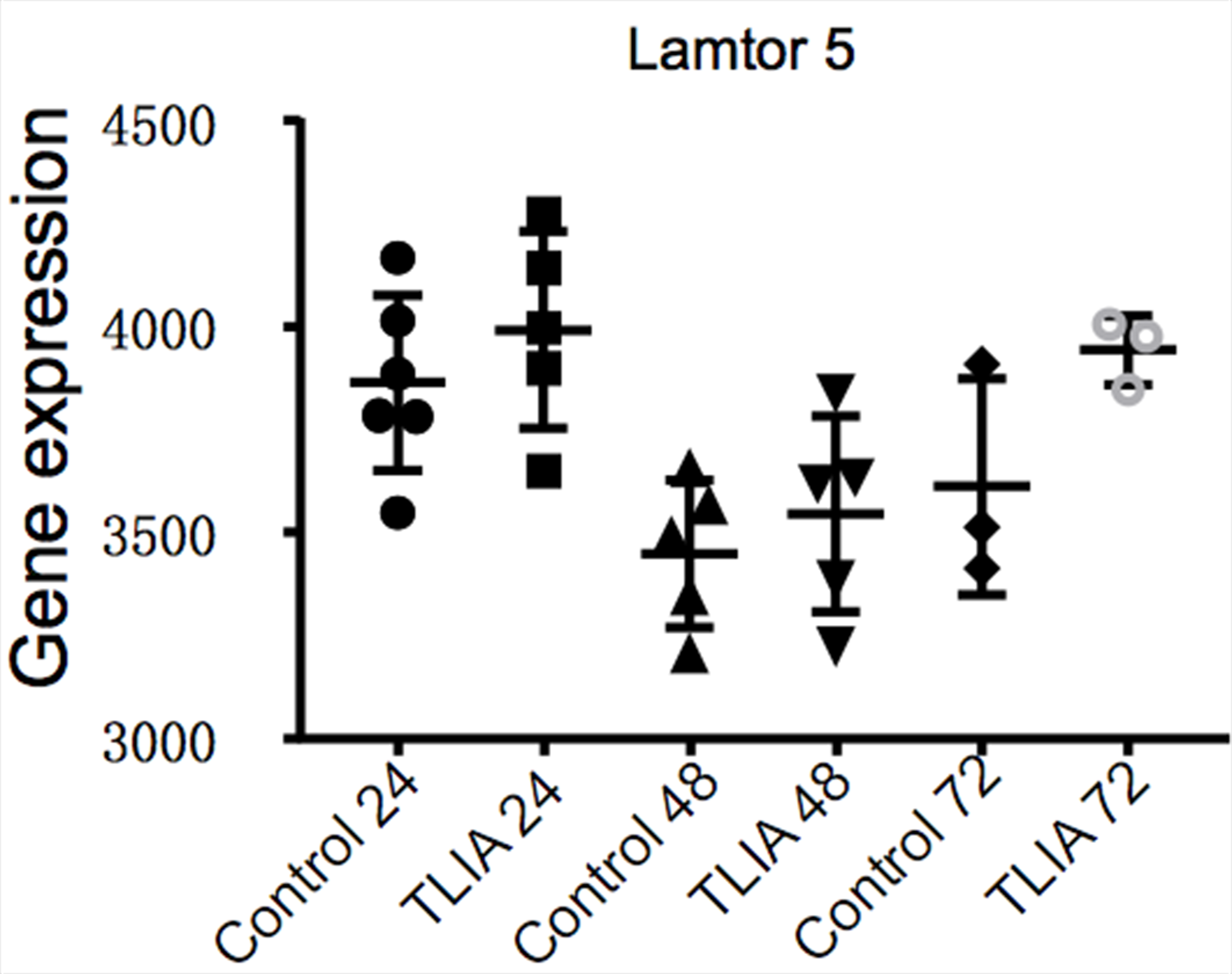

Supplement: Supplementary file 3 — Figure S3 [file 41419_2020_2362_MOESM3_ESM.tif]
