# Supplementary material for: B cells control lupus autoimmunity by inhibiting Th17 and promoting Th22 cells
Source: Cell Death Dis. 2020 Mar 3;11(3):164. doi: 10.1038/s41419-020-2362-y (PMC7054432; doi:10.1038/s41419-020-2362-y)
Supplement: Supplementary file 4 — Supplementary figure legend [file 41419_2020_2362_MOESM4_ESM.docx]

**Figure S1. B cells do not affect other subsets of T cell differentiation.** Naïve B cells isolated from B6 mice were cultured in the presence of α-IgM and α-CD40 for 2 days, then co-cultured with sorted naïve T cells in Th1 cell culture condition for 5 days. **(A)** CD4^+^IFN-γ^+^ cells were analyzed by flow cytometry (left). The results for flow cytometry of CD4^+^IFN-γ^+^ cells (right). **(B)** CD4^+^T-bet^+^ cells were analyzed by flow cytometry (left). The results for flow cytometry of CD4^+^T-bet^+^ cells (right). **(C)** Naïve B cells isolated from B6 mice were cultured in the presence of α-IgM and α-CD40 for 2 days, and then co-cultured with sorted naïve T cells in Treg cell culture condition for 5 days. IL-10 and TGF-β intracellular expression was analyzed by flow cytometry (left). The results for flow cytometry of IL-10^+^TGF-β^+^ cells (right). Results shown are representative of three independent experiments.

**Figure S2. Cytokine production in activated B cells.** Naïve B cells isolated from B6 mice were cultured in the presence of α-IgM and α-CD40 for 2 days. **(A)** TNF-α, IFN-γ, IL-10, TGF-β, IL-4, and IL-6 intracellular expression in CD19 cells were analyzed by flow cytometry. **(B)** TNF-α, IFN-γ, IL-10, TGF-β, IL-4, and IL-6 in supernatants were analyzed by ELISA. **(C)** IgM, IgG, and IgA in supernatants were analyzed by ELISA. Results shown are representative of three independent experiments. *, *p* < 0.05;**, *p* < 0.01; ***, *p* < 0.001.

**Figure S3. Expression of the mTOR activator LAMTOR5 in human Th17 cells.** Gene expression data in Gene Express Omnibus, GSE89133 was explored to show that human Th17 cells treated with TNF family member TL1A, with mTOR activator LAMTOR5 detected.
